# Photosystem I integrated into mesoporous microspheres has enhanced stability and photoactivity in biohybrid solar cells

**DOI:** 10.1016/j.mtbio.2021.100122

**Published:** 2021-07-09

**Authors:** Alexandra H. Teodor, Lucas B. Thal, Shinduri Vijayakumar, Madison Chan, Gabriela Little, Barry D. Bruce

**Affiliations:** a Program in Genome Sciences and Technology, Oak Ridge National Laboratory and University of Tennessee, Knoxville, USA; b Department of Biochemistry & Cellular and Molecular Biology, University of Tennessee, Knoxville, USA; c Department of Chemical and Biomolecular Engineering, University of Tennessee, Knoxville, USA; d Department of Engineering Management, University of Tennessee, Chattanooga, USA

**Keywords:** Microsphere, Photosystem I, Lyophilization, Biohybrid solar cell, Encapsulation

## Abstract

Isolated proteins, especially membrane proteins, are susceptible to aggregation and activity loss after purification. For therapeutics and biosensors usage, protein stability and longevity are especially important. It has been demonstrated that photosystem I (PSI) can be successfully integrated into biohybrid electronic devices to take advantage of its strong light-driven reducing potential (−1.2V vs. the Standard Hydrogen Electrode). Most devices utilize PSI isolated in a nanosize detergent micelle, which is difficult to visualize, quantitate, and manipulate. Isolated PSI is also susceptible to aggregation and/or loss of activity, especially after freeze/thaw cycles. CaCO_3_ microspheres (CCMs) have been shown to be a robust method of protein encapsulation for industrial and pharmaceutical applications, increasing the stability and activity of the encapsulated protein. However, CCMs have not been utilized with any membrane protein(s) to date. Herein, we examine the encapsulation of detergent-solubilized PSI in CCMs yielding uniform, monodisperse, mesoporous microspheres. This study reports both the first encapsulation of a membrane protein and also the largest protein to date stabilized by CCMs. These microspheres retain their spectral properties and lumenal surface exposure and are active when integrated into hybrid biophotovoltaic devices. CCMs may be a robust yet simple solution for long-term storage of large membrane proteins, showing success for very large, multisubunit complexes like PSI.

## Graphical abstract

Membrane proteins represent >25% of most genomes. The largest membrane protein with a known structure is photosystem I (PSI), which is of interest for biotechnological applications for its photogenerated reducing potential. Most membrane proteins are isolated using detergents, rendering them prone to activity loss and degradation. We report a method for stabilization of PSI in uniform, non-aggregating mesoporous microspheres based on CaCO_3_ templates with retained photochemical activity and improved stability and activity when incorporated in biohybrid photovoltaic devices.

**Figure:**
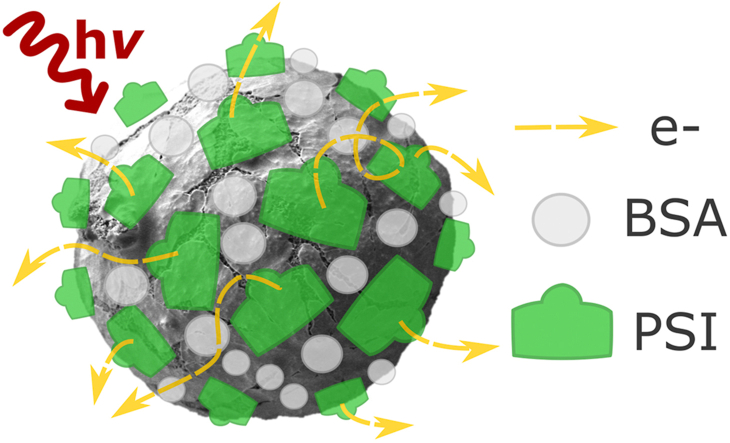

## Introduction

Recombinant or purified proteins have been utilized in many applied areas of biotechnology including pharmaceutical use, industrial enzymatic applications, and more recently biohybrid devices [[1], [2], [3]]. However, numerous issues must be overcome for successful integration of any biomaterial, including proteins, for biotechnological applications. These issues include decreased stability outside of the cellular environment, loss of activity, difficulties in manipulating/engineering the bio/inorganic interface, and the low molecular weight and size of most proteins making them difficult to quantitate and disperse uniformly.

The use of microspheres, specifically CaCO_3_ microspheres (CCMs), as stabilization matrices for therapeutic and industrial use has been previously documented [[4], [5], [6]]. CCMs are of particular interest to research communities because of (i) a facile and inexpensive fabrication process, (ii) the large degree of control over their properties to generate uniform particles with diameters in the nano- to micro-meter range, and (iii) the material is biologically inert and non-toxic [7]. CCMs also have mild conditions for decomposition and removal of the template while preserving the encapsulated biological material after cross-linking, along with high porosity and surface area making them even more attractive for applications where catalytic activity enhancement is desired [6,8]. Herein, we describe a fabrication protocol yielding uniform, non-aggregating CCM templates for the encapsulation of membrane proteins, specifically the protein-pigment complex, photosystem I (PSI).

PSI is one of the two multisubunit protein-pigment complexes, known as reaction centers, central to the light reactions of oxygenic photosynthesis [9]. The other reaction center central to oxygenic photosynthesis, photosystem II (PSII), has previously been studied using a CCM template coated in a proteoliposome with ATPase present for ATP production [10]. However, PSII is remarkably unstable and is known to degrade after approximately only a dozen photoexcitation events, and the authors of this study did not investigate the stability of their system or long-term storage possibility. These reaction centers are responsible for the conversion of light into high-energy electrons needed for downstream carbon fixation in most photosynthetic organisms. PSI in most photosynthetic organisms comprises approximately 12–15 protein subunits, 100 chlorophyll molecules, 20 carotenoids, 2 phylloquinones, and 3 Fe_4_S_4_ clusters and even takes on higher oligomeric states, putting it among the most complex protein complexes in Nature and reaching sizes of 1.2 MDa for its trimeric form [[11], [12], [13], [14], [15], [16], [17]]. PSI generates the lowest reducing potential in biological electron transfer (−1.2 V vs. SHE) on photoexcitation, catalyzing an electron transfer across the reaction center from its lumenal to its stromal face [3]. PSI, in particular, has been of interest for the development of biohybrid technologies [[18], [19], [20], [21]], particularly photovoltaic devices based on dye-sensitized solar cells [2,[22], [23], [24], [25]], with current densities in PSI-based photovoltaic devices improving over 6 orders of magnitude over the last decade and continuing to rise [3,[26], [27], [28], [29]]. After isolation and incorporation into biohybrid devices, PSI shows no significant loss in activity for over 90 days for cyanobacterial and at least 280 days for higher plant PSI [30,31]. Furthermore, the most commonly used biological sources of PSI for biotechnology applications, *Thermosynechococcus elongatus* BP-1, *Chlamydomonas reinhardtii,* and *Spinacea oleracea*, are all amenable to genetic modification of their reaction centers to engineer in desirable characteristics [32]. These many properties of PSI make it of great interest for use in biotechnology applications. Yet, PSI as a membrane protein-pigment complex is susceptible to degradation under unfavorable conditions and is unable to withstand freeze-thaw cycles or lyophilization without aggregation and/or loss of activity. Furthermore, the relatively small size of PSI and other membrane proteins are hindrances for ease of handling for biotechnology applications, and the ability to generate larger superstructures such as microspheres will allow for greater ease of handling and manipulation, such as addition or removal from reaction vessels, microfluidics devices, or novel electrode architectures [[33], [34], [35], [36]]. However, many researchers in this field have focused on studying novel electrode materials and architectures including mesoporous inverse opal configurations to improve device performance and outputs [37]. Multiple strategies that have been proposed for stabilization of the protein component for biohybrid devices are based on the use of surfactant peptides. Previous work on engineering poly lactic-*co*-glycolic acid (PLGA) microparticles with PSI was shown to help keep PSI stable and retained photochemical properties [38]. These microparticles, similar to CCMs, provide a protective three-dimensional matrix to stabilize PSI from the outside environment. However, PLGA is relatively expensive, and picking the right polymer by average size and chemical functionalization for a protein of interest may be difficult and may affect protein activity in unknown ways [39].

Herein, we utilize CCMs as a method of generating a three-dimensional template that has embedded PSI and a passive bridging protein, bovine serum albumin (BSA). After precipitation of the CaCO3 as a micron-sized spherical template, the two proteins are chemically cross-linked together with glutaraldehyde, forming intermolecular and intramolecular covalent linkages between the surface exposed amino acids of PSI and BSA. Glutaraldehyde is one of the most widely used bifunctional cross-linking reagents [40]. After cross-linking, the CaCO_3_ template is chelated with addition of ethylenediaminetetraacetic acid (EDTA), a commonly used chelation agent for removal of 2^+^ cations. After the CaCO_3_ template is chelated away, it leaves relatively similar sized, mesoporous proteinaceous microspheres with an aqueously accessible interior. After fabrication, it is expected that the PSI encapsulated in microspheres is in a stable state similar to free PSI while also maintaining its photochemical properties while being immobilized into comparatively large and uniform microspheres that are easy to visualize and manipulate. To test PSI microsphere stability and performance as well as measure various parameters, particles were tested by low temperature fluorescence, pump-probe spectroscopy, dynamic light scattering (DLS), as well as SEM imaging and UV-vis spectroscopy. We also incorporated PSI microspheres into a biophotovoltaic device and report obtained photocurrents, showing the utility of the stabilized protein for *in vitro* applications.

## Materials and methods

### PSI purification and microsphere synthesis

Salt solutions of 0.05 M sodium carbonate (NaCO_3_), 0.5 M calcium chloride (CaCl_2_), BSA at 10 mg/mL in MES buffer, PSI at 1 mg/mL chlorophyll *a* in MES buffer with 0.03% dodecyl maltoside (DDM), and 0.05 M MES buffer at pH 6.4 with 0.03% DDM were used for synthesis. PSI was isolated and purified as described by Iwuchukwu et al. [30]. Solution ‘A’ was composed of 400 μL CaCl_2_, 100 μL BSA, and 28 μL PSI. Solution ‘B’ was composed of 400 μL NaCO_3_, 100 μL BSA, and 28 μL PSI. Solution ‘A’ was pipetted into solution ‘B’ while being vortexed at lowest speed on a Fisher vortexer. An apparatus was constructed to help stabilize the microcentrifuge tube while the solutions were being mixed. The newly made microspheres are then centrifuged at 1500×*g* for 2 min. The supernatant was then removed, and the microsphere pellet was washed using MES buffer. This process of centrifugation and washing was then repeated 2 more times. After the final wash, the supernatant is removed, and a solution of 0.025% glutaraldehyde in MES buffer is added and then left with gentle stirring for 2 h to cross-link the proteins within the template CCM. After this, 0.25 M glycine is added to quench the cross-linking process. Once the microspheres are quenched, they are centrifuged and washed three times using MES buffer. The sample is then resuspended in EDTA on the final wash and left to chelate overnight; this process helps remove the CaCl_2_ template around each microsphere. PSI concentration was kept constant for varying protein composition samples, and BSA was increased accordingly.

### Size distribution analysis

Size distribution analysis was performed using template microspheres made using BSA, Na_2_CO_3_, CaCl_2_, and MES buffer. Once the microspheres were combined and vortexed, they were let to sit for varying amounts of time from 0 s to 300 s before being centrifuged and washed. Samples were then taken and put onto glass slides to be imaged using a differential interference contrast (DIC) microscope. Image J analysis [41] was used to collect data on the size distribution on the microsphere sample. Images taken from the microscope were converted to grayscale 16 bit, and then the ‘threshold’ function was used to highlight the microspheres that needed to be counted. Diameter data from image analysis were then binned to form a histogram of particle size (bin size = 0.1 μm), which was then fit with a single Gaussian curve using GraphPad Prism 8 (1000 iterations, no constraints on fitting or outliers excluded). To determine whether protein composition (BSA:PSI ratio) had an effect on the size of synthesized microspheres, samples at ratios of 250:1, 500:1, and 1000:1 BSA:PSI were synthesized as described previously, and samples were run on a Wyatt Technologies DynaPro NanoStar DLS instrument. Samples were run to an n = 1,000,000 for accurate measurements of diameter with technical replicates n = 10 for each sample.

### UV-vis spectroscopy

UV-vis spectroscopy was used to measure the absorbance of PSI in the microspheres. Samples were transferred to cuvettes, and a blank of MES buffer was used. Samples were run on a Thermo V-Vis Evolution 300 Spectrophotometer. Samples were normalized to maximum absorbance values measured, with scattering from the template (non-protein containing CCMs formed at the same salt concentrations listed previously) subtracted out as a baseline.

### Scanning electron microscopy (SEM)

Samples were prepared as described previously at varying concentrations and aliquoted at various points of the synthesis process. The samples were rinsed and resuspended in deionized water before imaging. Samples were then imaged on the Zeiss Auriga Crossbeam FIB/SEM at EHT = 2 kV under vacuum after sputter-coating with Au to improve image quality.

### Low-temperature fluorescence emission spectroscopy

Samples were loaded into EPR tubes and flash-frozen by lowering into a vacuum-insulated Dewar flask which was filled with liquid N_2_. Each tube was slowly immersed to allow for full freezing. Microsphere samples were taken at three stages during the synthesis for quantification of PSI stability: before cross-linking, after cross-linking, and after chelation. Samples were also synthesized and quantified at three BSA-to-PSI ratios: 250:1, 500:1, and 1000:1. Samples were measured using a PTI QuantaMaster dual-channel fluorometer (PTI/Horiba, NJ). Samples were excited at a wavelength of 430 nm and a slit width of 0.75 nm. Emission was then acquired from 600 to 800 nm. Three replicate spectra were acquired for each sample.

### Laser scanning confocal microscopy (LSCM)

LSCM of PSI chlorophyll autofluorescence was analyzed using a Leica TCS-NT laser confocal microscope. Chlorophyll autofluorescence was visualized using 488 nm excitation and a 660 LP filter (chlorophyll autofluorescence). A 100 × 1.4 NA oil immersion lens at an Airy disc setting of 0.91 was used. The microspheres were vertically sectioned using Z = steps of 60 nm. The fluorescence intensity cross-section was derived directly from image analysis of each Z-slice using ImageJ (Ver. 1.53).

### Flow cytometry

Samples were prepared as described previously at BSA:PSI ratios of 250:1, 500:1, and 1000:1 with PSI concentration fixed. Samples were cross-linked and chelated for fluorescence-activated single-cell sorting. Chlorophyll autofluorescence (excitation at ~480 nm, emission at ~690 nm using PerCP tracking) was used for microsphere detection in the instrument. Samples were run on a BD LSR II flow cytometer (BD Biosciences) to 50,000 counts per sample, repeated in biological duplicates. Data were evaluated using FlowJo Mac software, version 10.1.

### Pump-probe spectroscopy

Pump-probe spectroscopy was used to assess the ability of the microspheres to act as electron donors. Spectroscopy was performed using a SpectroLogiX JTS-100, using the Bio-Logic USA P700 detection LED lamp which measures absorbance changes at 705 nm for tracking PSI reduction. Excitation was performed using the built-in actinic source at 630 nm. The pulse sequence used as defined in the JTS software was 20(D50 ms)G[150000 μA]30 msHT10 μs{20 μs,400,5000 ms,D}. Addition of the electron donor, 2,6-dichlorophenolindophenol (DCPIP), was titrated in to PSI at different concentrations noted in figures. Final chelated samples of PSI were diluted 2:5 in 0.05 M MES buffer at pH 6.4 with 0.03% DDM. Methyl viologen was added to a concentration of 1 mM as a sacrificial electron acceptor and ascorbate to 1 mM as a sacrificial low level for reduction of PSI on the many minutes' timescale to ensure PSI in the dark was fully reduced. A 1 s baseline was taken before the actinic source was turned on for 30 ms to fully photo-oxidize the PSI present, and the reduction in the dark was tracked for 5 s using an exponential clock. Five traces per sample were averaged. Monophasic exponential association curve fitting with 1000 iterations in Prism 8 (GraphPad Software, San Diego, CA) was used to fit the reduction data to a single exponential equation to calculate observed reduction rates (K_obs_, units ms^−1^). Linear fitting of K_obs_ vs. [DCPIP] was performed using simple linear regression in Prism 8 (GraphPad Software, San Diego, CA).

### Biophotovoltaic device fabrication and testing

Indium Tin Oxide (ITO)-coated conductive glass electrodes (~25 mm^2^) were doctor-bladed with Solaronix T/SP TiO_2_ nanoparticle paste to deposit a transparent mesoporous semiconductor layer on the conductive side of the electrode. Electrodes were then sintered after a ramp up from 23 °C to 475 °C and held for 30 min at 475 °C before allowing cooling. Electrodes were stored in airtight containers with desiccant before use. PSI microspheres with a BSA:PSI ratio of 250:1 were synthesized and chelated overnight before being drop coated onto an ITO/TiO_2_ electrode. Counter electrodes were generated by carbon deposition on the conductive side of the electrode. The 2 electrodes were then offset and mechanically compressed together, with dual clamps on either side holding the device together. The liquid junction electrolyte using an aqueous bipyridine-based Co(II/III) redox mediator at a concentration of 10 mM was then introduced, prepared as described previously in the same MES buffer used throughout analysis with 0.03% DDM [42]. Devices were allowed to sit for approximately 30 min to allow for electrolyte integration before device testing. Device illumination was performed using a Schott KL-2500 light source with an inset filter holder and Schott red light filter (MOS-258-303) for all red actinic light experiments. All experiments were performed at the open circuit potential (no applied bias). All photochronoamperometric measurements were taken using a Bio-Logic SP-50 potentiostat and EC-Lab software for data collection. Data plotting was carried out in Origin 2020 and GraphPad Prism 9.

## Results

**Fig. 1:**
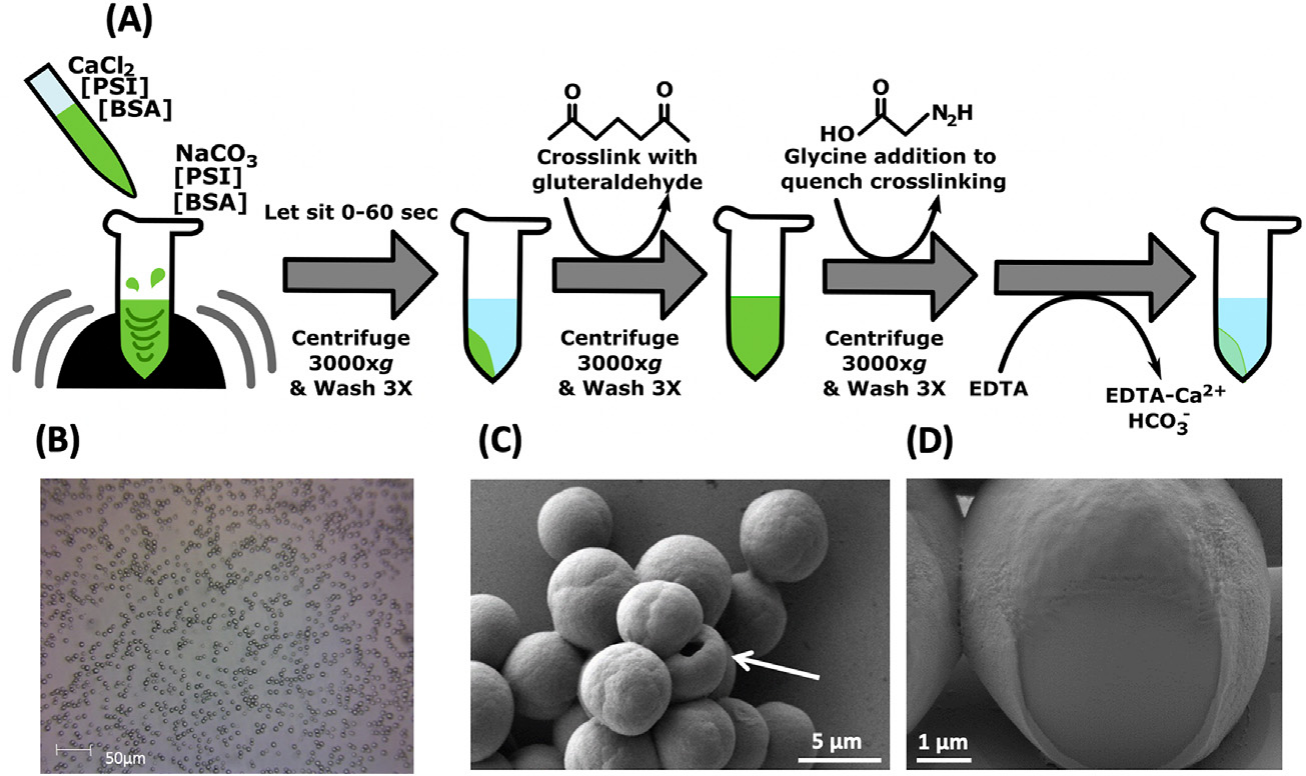
**Fabrication of mesoporous PSI microspheres.** A. The fabrication protocol for uniform, dispersed microspheres showing the change in appearance before the CaCO_3_ template is removed by chelation. B. The DIC image of fabricated template microspheres with no PSI, showing their monodispersion and uniform size. C. The scanning electron microscopy (SEM) image of a cluster of PSI-containing microspheres at a ratio of 500:1 BSA:PSI at the pre–cross-linking stage shows the smooth CaCO_3_ template surface. A rare hollow microsphere is pointed out with a white arrow. D. Focused ion beam dissection of the same pre-chelation sample in 1C reveals that the microspheres are solid and uniform throughout.

We have developed a simple, scalable, easily modifiable fabrication method for highly uniform, non-aggregating CCMs that have the capacity for membrane proteins to be readily incorporated and accessible to substrates, i.e. redox mediators or catalytic substrates. A functional protein, such as PSI, can be incorporated along with a bulk, inert filler protein such as BSA to help further stabilize the microsphere. To best illustrate the structure of these PSI-based microspheres, a brief diagram of the fabrication protocol is given in Fig. 1A, with the complete fabrication protocol given in the Materials and Methods. This protocol helps to minimize alternative crystallization patterns of the CaCO_3_ template, allowing for formation nearly exclusively of the spherical vaterite crystal pattern and very little of the cubic calcite crystal pattern.

The uniformity of particles produced can be seen easily by light microscopy even at relatively low magnifications as shown in the DIC microscopy in Fig. 1B, where it is also clear that the particles are well dispersed and are not prone to self-association or aggregation, making them easier to observe, characterize, and manipulate. Further investigation at much higher magnification, by scanning electron microscopy (SEM), revealed the smooth surface of the CCM template surface before overnight chelation, in Fig. 1C. Focused ion beam dissection of the same microsphere sample from 1C shows that the microspheres produced are typically solid and uniform throughout, although there are rare cases of hollow particles forming, as seen in Fig. 4F.

**Fig. 2:**
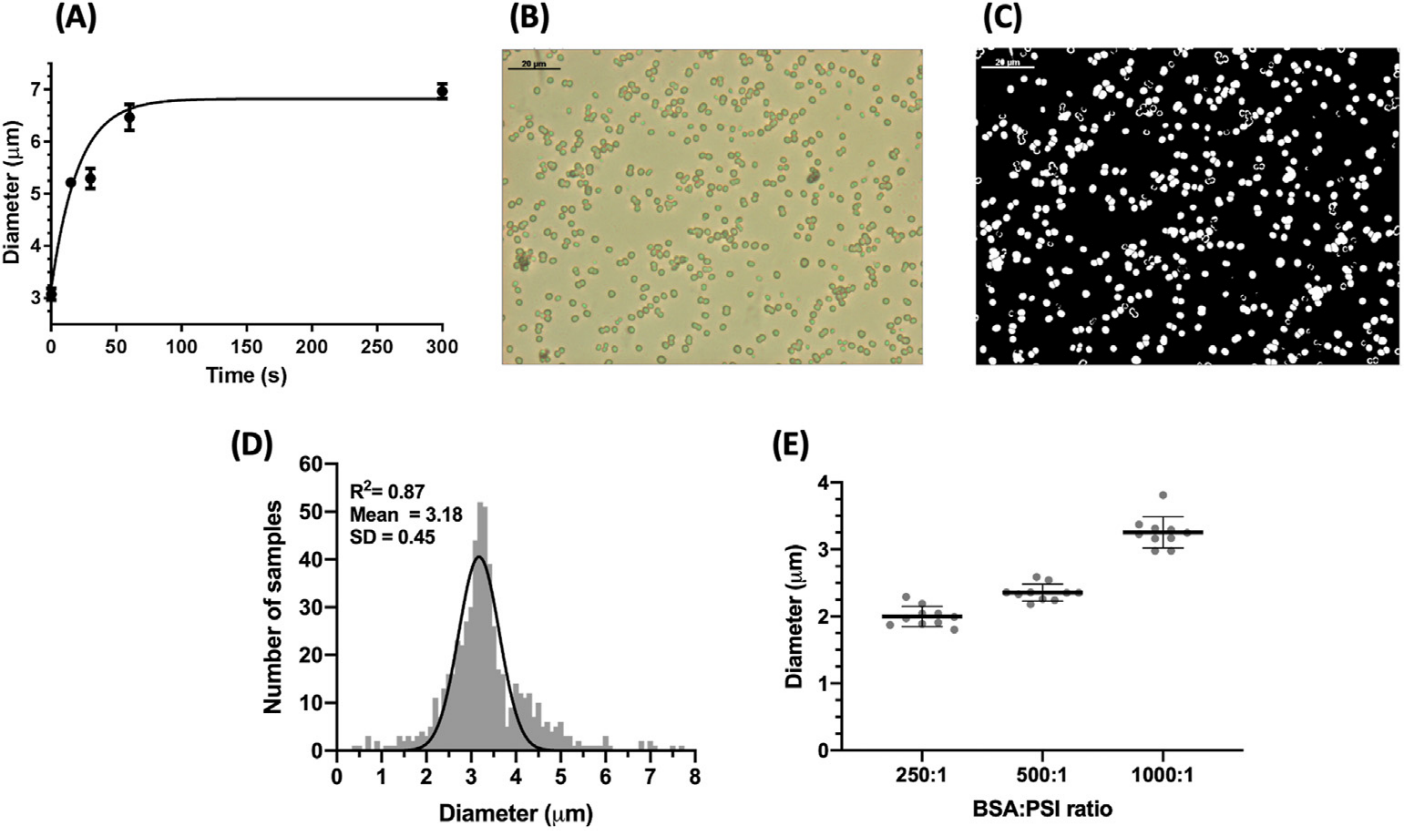
**Control of PSI microsphere characteristics during the fabrication process. A.** Size of microspheres can be controlled during the fabrication process as a function of time. BSA-only–containing template microspheres were fabricated and allowed to sit for various times immediately after initial vortexing. Size was measured via differential interference contrast (DIC) microscopy image analysis using ImageJ. **B.** A representative DIC microscopy image used for size analysis of PSI-containing microspheres after chelation, BSA:PSI ratio 500:1 and initial particle incubation of ~5 s. **C.** A representative image analysis showing the particles analyzed using ImageJ macros for size analysis measurement. All white particles are those automatically detected by the software and analyzed for shape and size. Samples here and analyzed in panel D contain only BSA protein. **D.** A histogram showing the size distribution of the particles in **C** including other images of the same sample, n = 576; bin size = 0.25 μm. **E.** The inclusion of PSI in template microspheres at increasing concentration (lower BSA:PSI ratios) leads to decreased diameter of microspheres after chelation. The diameter of particles was measured using DLS. The data represent 10 different full measurements of DLS of each sample, each to an N = 1,000,000.

**Fig. 3:**
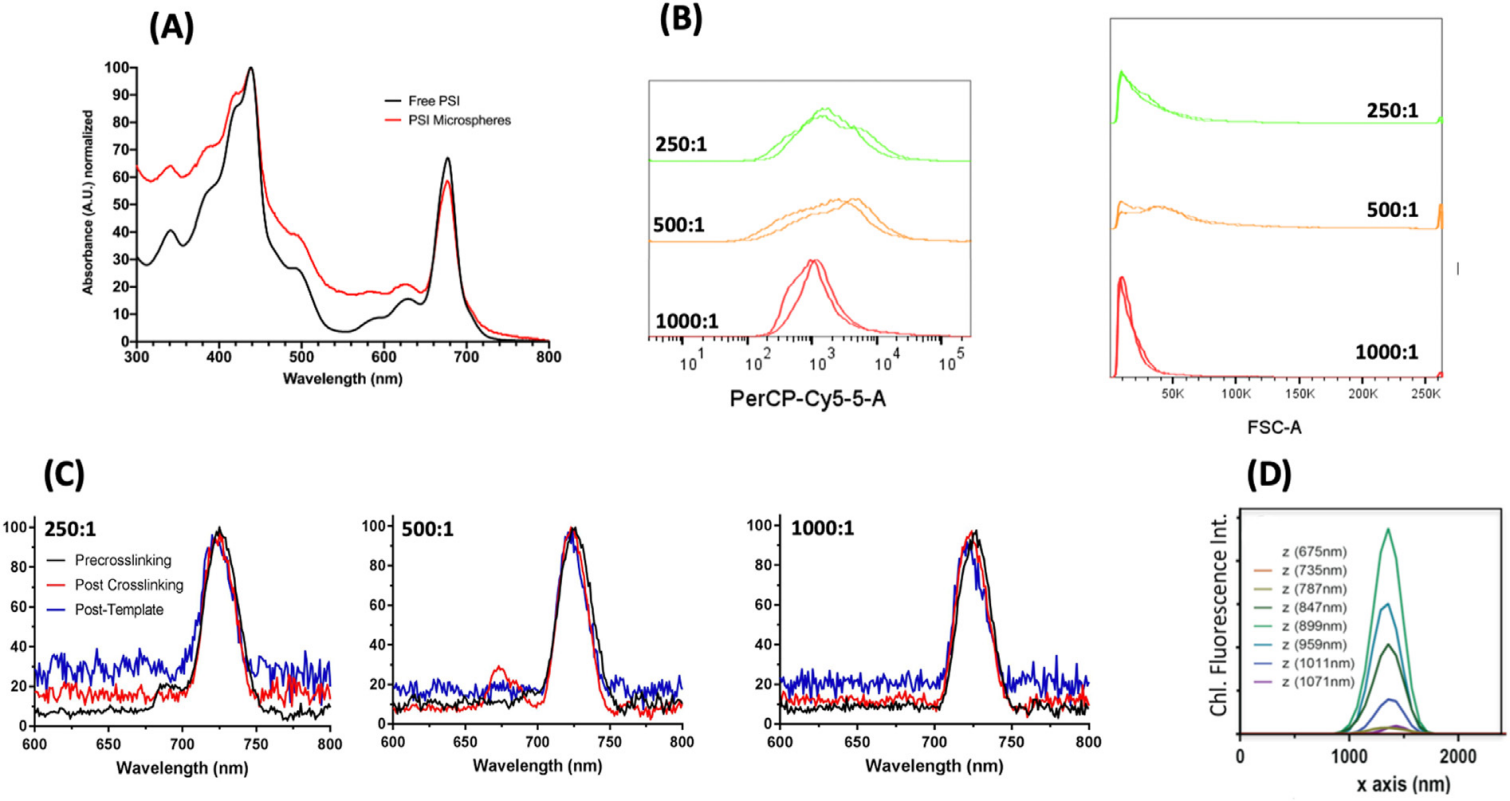
**Incorporation of PSI into template CaCO**_**3**_**microspheres is stable and uniform. A.** Room temperature UV-vis absorbance spectra of free PSI as compared with PSI encapsulated in microspheres are shown. Free PSI is shown in black, and PSI microspheres with template BSA-only microsphere scattering subtracted are shown in red. Spectra were normalized to their maximum absorbance values. Microspheres were fabricated with a BSA:PSI ratio of 500:1. **B.** Flow cytometry of PSI-containing microspheres. Ratios indicate molar ratio of BSA to PSI. Two replicate sets of samples were run at n = 50,000 for accurate comparison. The panel on the left shows fluorescence distribution of PSI embedded within the particles due to chlorophyll autofluorescence. **C.** Low-temperature fluorescence of PSI microspheres. Ratios indicate the molar ratio of BSA to PSI. Samples were assessed at three stages of the microsphere fabrication process to assess the stability of encapsulated PSI during the fabrication process. PSI has a characteristic 77K fluorescence emission peak at 720 nm, and free chlorophyll has a characteristic emission peak at 660 nm. **D.** Z-stack confocal fluorescence microscopy of PSI microspheres at a ratio of 500:1 reveals uniform chlorophyll fluorescence throughout the microsphere.

**Fig. 4:**
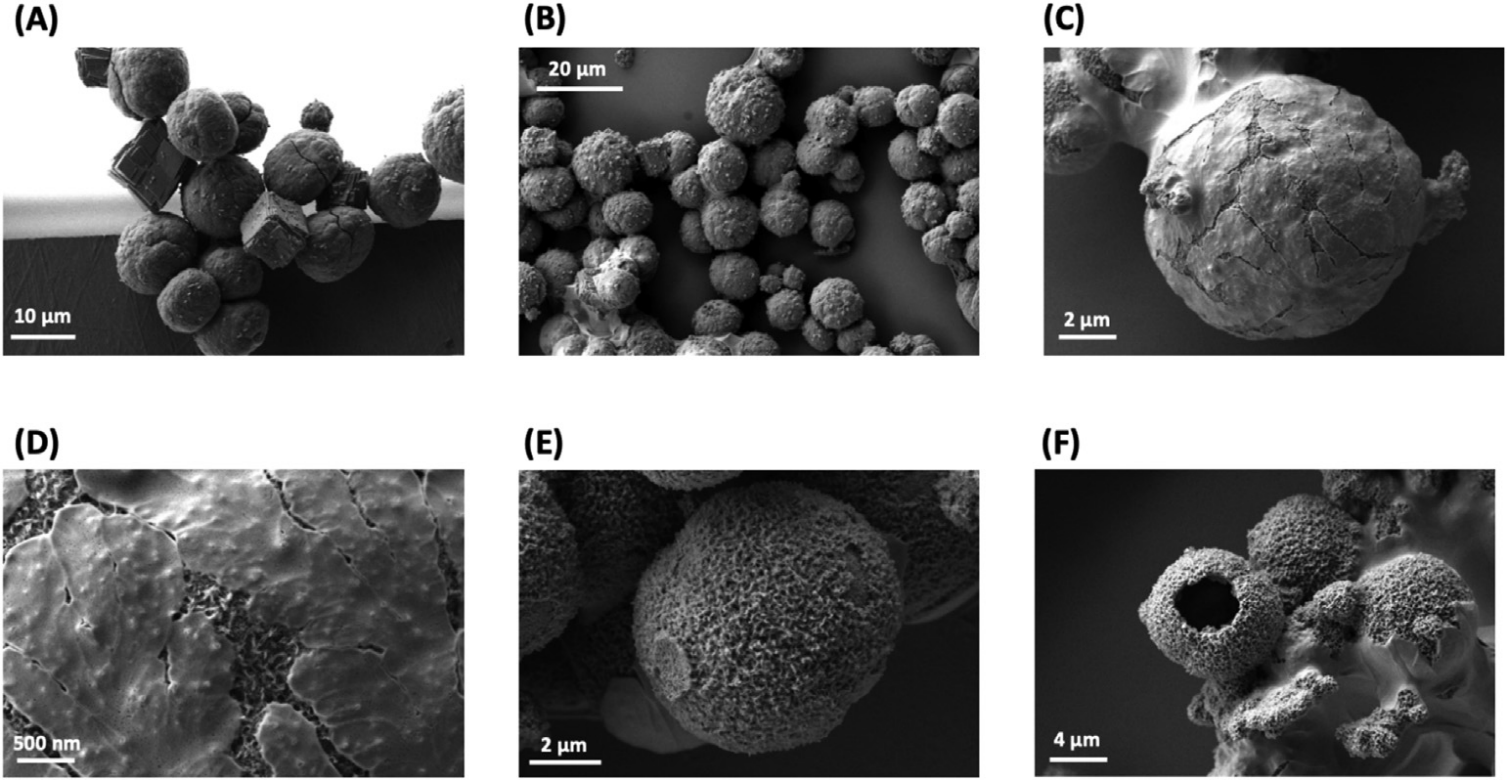
**Electron micrographs of PSI-containing microspheres reveal surface characteristics.** All images were captured using the Zeiss Auriga Dual Beam FIB/SEM, and all samples were sputter-coated with Au before imaging. All samples were at a BSA:PSI ratio of 500:1. **A.** An electron micrograph showing the difference between cubic calcite and spherical vaterite CaCO_3_ particles. **B.** PSI-containing microspheres before cross-linking. **C.** PSI-containing microspheres after cross-linking. **D.** A close-up of the same sample from **D** showing the difference in texture between the CaCO_3_ template and the protein encased. **E.** PSI-containing microspheres after chelation of the CaCO_3_ template. **F.** An example of a rare hollow microsphere after chelation. Nearly all microspheres are solid throughout as shown in Fig. 1.

The ability to control the physical properties and characteristics of these proteinaceous microspheres during the fabrication protocol is key in changing their properties to better suit specific applications and uses. In this work, we use BSA as a benign, space-filling protein to increase the number of protein cross-linking sites between the two macromolecules. There are multiple methods for controlling the characteristics of produced microspheres [43,44]. However, by varying either the MW of this space-filling protein or the relative protein concentrations of the two starting protein solutions highlighted in Fig. 2, microsphere characteristics such as size can be controlled as shown in Fig. 2E. Other researchers have noted that other characteristics such as porosity and rigidity of the produced CCMs can also be controlled by other factors of the synthesis process [45]. By varying the length of precipitation time the particles are allowed to ‘grow’ during this initial period before the first washing to remove excess Ca^2+^ and CO_3_^2−^ ions present in the solution, it is possible to control the diameter and size of the particles produced. We found increases in size of over 200%, from approximately 3 μm which formed very rapidly (<5 s) on mixing the Ca^2+^ and CO_3_^2−^ ions to approximately 7 μm in particle diameter after 5 min. The range of sizes observed in the pre-washed samples was analyzed via quantitative image analysis in Fig. 2A. The particle size quickly increased during the first 75 s before reaching a relatively stable plateau in particle diameter. However, by quenching the reaction by dilution, it is possible to achieve relatively uniform particles after a defined time. The size distribution of these particles is shown by light microscopy and image analysis in Fig. 2B and C. The analysis of these particles was performed using ImageJ software to detect, measure, and allow graphing of the size distribution shown in Fig. 2C. By performing analysis on multiple images to get a larger sample size (N > 500 particles), we observed a Gaussian distribution of particle size centered on 3 μm as predicted from initial testing results. One additional factor that was found to affect the size of the fabricated microspheres was the ratio of BSA filler protein to PSI in the incorporated protein microparticles. The concentration of PSI used was kept constant while BSA was increased to fabricate particles with BSA:PSI ratios of 250:1, 500:1, and 1000:1. As the ratio of BSA:PSI increased and overall protein concentration increased, the diameters of the microparticles produced similarly increased in size, with an increase of over 1 μm in size noted going from the 250:1 to the 1000:1 ratio.

To test whether PSI is destabilized during microsphere synthesis, the incorporation of PSI into the template CCMs was more closely investigated via UV-vis spectroscopy (Fig. 3). Because of the large abundance of chlorophyll present bound in PSI that functions as a light-harvesting antenna, PSI has a significant and characteristic absorbance pattern in the UV-visible region of the electromagnetic spectrum. After washing the particles after chelation to remove the CaCO_3_ template, the absorbance of PSI-containing microspheres was compared with free PSI, the spectra of which can be seen in Fig. 3A. Spectra of the free and microsphere-bound PSI were quite similar, with some increased absorbance from the PSI bound in microspheres, indicating that chlorophyll was successfully captured and cross-linked into the fabricated microspheres.

To further assess the presence of chlorophyll and uniformity of PSI incorporation in microspheres, flow cytometry was performed, the results of which can be seen in Fig. 3B. This method allows tens of thousands of particles to be quickly analyzed to build a statistically significant histogram of both the chlorophyll fluorescence and the light scattering properties. These two parameters reflect the amount of PSI integrated per particle, and the light scattering yields a signal that is directly proportional to the particle diameter. Interestingly, microspheres formed at a BSA:PSI ratio of 1000:1 were found to have the most uniform chlorophyll fluorescence (PerCP 695/40 emission) (Fig. 3B) as well as tightest size distribution based on forward angle light scattering in Fig. 3C. This suggests that the ratio of filler protein to protein of interest plays a role not only in the size of microspheres but also in the degree of incorporation of the protein of interest in the final product. The uniformity of chlorophyll incorporation in PSI-containing microspheres was further confirmed by three-dimensional confocal fluorescence microscopy as seen in Fig. 3D, revealing maximal fluorescence was collected at the center z-slice, as well as consistent Gaussian emission spectra throughout the z-stack.

While flow cytometry and UV-vis absorbance spectroscopy are both informative on the micron scale, PSI is a complex multisubunit protein complex with many internal cofactors bound, and a complete characterization requires these data to be complemented with nanoscale observations and activity assessment. To assess the integrity of the PSI complex captured in microspheres, low-temperature fluorescence (LTF) spectroscopy was performed. Chlorophyll bound in its native conformation in PSI has a signature LTF spectrum compared with free chlorophyll, with PSI-bound chlorophyll producing a characteristic emission peak at 720 nm. The lack of additional peaks at ~665 and ~680 nm indicates very little free chlorophyll or uncoupled chlorophyll within the PSI complex. Samples of microspheres at BSA:PSI ratios of 250:1, 500:1, and 1000:1 were assessed by LTF at three separate stages of the fabrication process, results of which are shown in Fig. 3C. All samples at all BSA:PSI ratios showed the peak characteristic of chlorophyll bound stably in its native conformation in PSI, with a small amount of free chlorophyll present in the 500:1 post–cross-linking sample. These results suggest that the CCMs act as a stable template for membrane protein complexes such as PSI with minimal loss or disruption of the chlorophyll pigments during fabrication.

After investigating the size distribution and uniformity of the fabricated particles, the surface characteristics were next investigated through the use of SEM. Representative electron micrographs captured of samples are shown in Fig. 4. Fig. 4A shows the difference in crystallization patterns between the vaterite and calcite crystallization patterns of the CCM template, generating spherical and cubic particles, respectively. An image of a sample of PSI-containing particles before cross-linking can be seen in Fig. 4B. After cross-linking, the particles can be seen in Fig. 4C with distinct delineations between the CaCO_3_ template appearing smoother on the surface and the PSI and BSA proteins included in the microsphere appearing rougher underneath the surface. Fig. 4D is a zoomed-in view of the same sample in 4C and more clearly shows the difference in surface texture between the CaCO_3_ salt template and the rougher proteinaceous surface. In Fig. 4E, samples were imaged after chelation and removal of the CaCO_3_ template, revealing the textured mesoporous surface of the proteinaceous microspheres. Finally, in Fig. 4F, another example of a rare hollow microsphere can be seen.

**Fig. 5:**
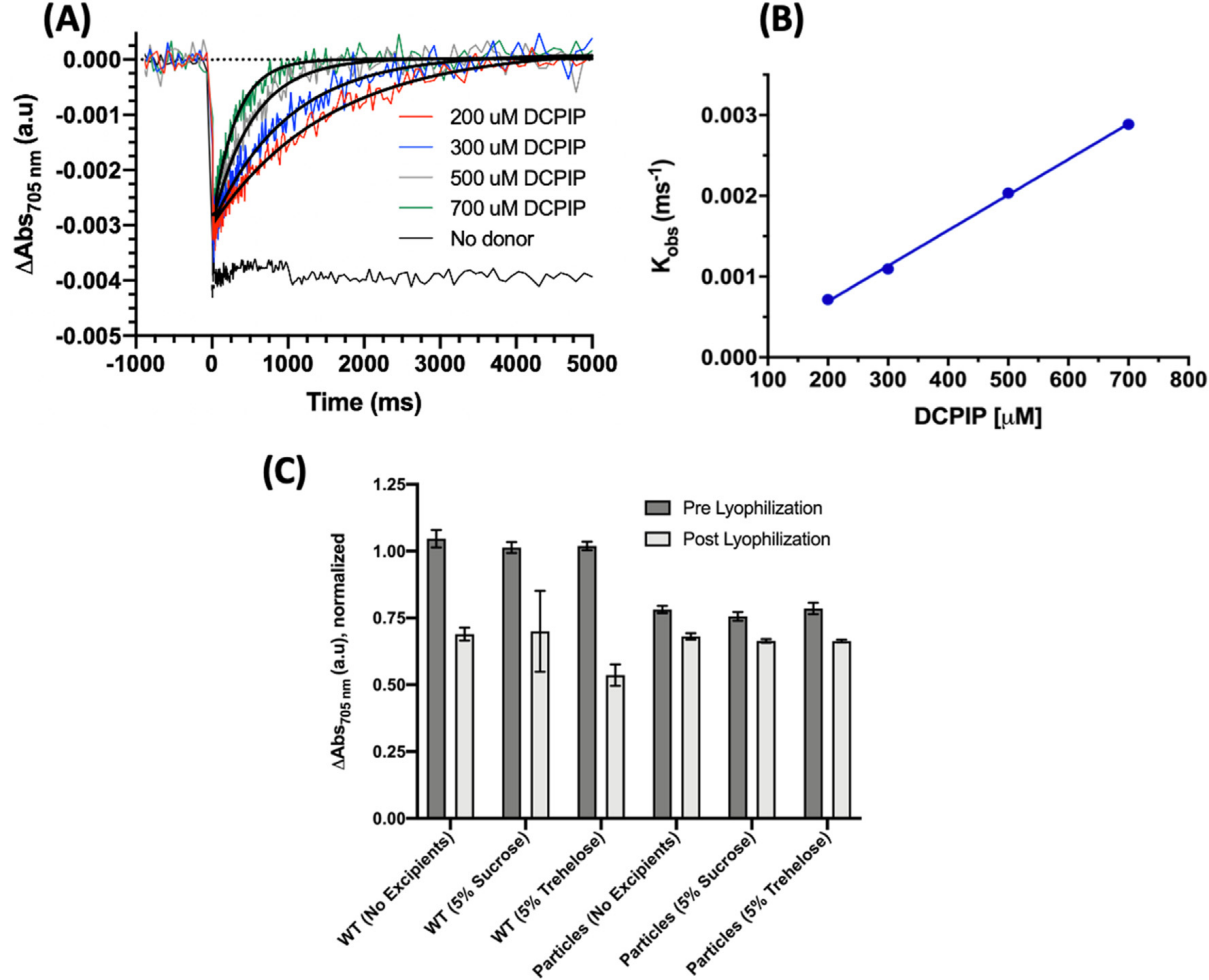
**PSI photoactivity is retained after incorporation into microspheres, and microspheres retain photoactivity after lyophilization.** Chelated microspheres containing PSI at a ratio of 500:1. **A.** Pump-probe spectroscopy was performed to assess PSI electron transfer capability after encapsulation in CaCO_3_ microspheres. **B.** Observed PSI reduction rates (K_obs_) are plotted against the concentration of the donor during titration. **C.** PSI microsphere photoactivity was assessed before and after lyophilization to test long-term stability and compared with WT (free) PSI. Excipients sucrose and trehalose at 5% w/v were also included to assess their effects on lyophilization stability.

To assess the photoactivity of the PSI-containing particles, pump-probe spectroscopy was performed. In pump-probe spectroscopy, the sample is excited with one beam, ‘pumping’ the sample, and the second beam ‘probes’ the sample to assess spectral changes of the sample at particular wavelengths. After photo-oxidation, no reduction of the sample was seen when there was no electron donor in the form of DCPIP present, shown in Fig. 5A. However, as DCPIP was titrated in at increasing concentrations while the 500:1 BSA:PSI microsphere concentration was kept constant, an increasing rate of reduction was similarly seen in Fig. 5A. The observed data were fit to a one-exponential association function to calculate observed rates (k_obs_), which were then plotted against the concentration of the donor for each measured sample. As demonstrated in Fig. 5B, a linear trend in the observed PSI reduction rate was observed. Furthermore, all traces of PSI titration with DCPIP showed that all PSI photoexcitation signal was reduced back to the baseline, suggesting that all of PSI present in the microspheres was accessible to the DCPIP reductant, otherwise the signal would plateau before reaching the baseline. Taken together, these results suggest that the activity of PSI is both retained and stable after incorporation into the CCMs. These results also reveal that after cross-linking and template removal, the synthesized microspheres remain porous for exogenous addition of small molecule substrates to diffuse through and access the entirety of the protein bound within, a beneficial characteristic for industrial applications where high surface area for greater reactivity is desired.

The use of CCMs for long-term storage of PSI was also tested to see if CCMs are a viable long-term storage solution for proteins of interest, the results of which are seen in Fig. 5C. PSI-containing microspheres were lyophilized overnight with either no excipients or with 5% sucrose or trehalose purposed as cryopreservation agents [[46], [47], [48], [49], [50]]. Once the lyophilized samples were resuspended in buffer, we tested the photoactivity retention of the particles by comparing the photo-oxidation magnitude of post-lyophilized samples to the pre-lyophilization control. Interestingly, we see a reduction of ~25% of the P700 photobleaching signal relative to the free PSI with or without excipients; we suspect that some of this may be due to the slow settling microspheres during the measurement. However, less than five percent of the photoactivity of all microspheres was lost during the lyophilization process. Even though PSI captured in CCM-templated microspheres has some reduction in photoactivity signal, the protein microspheres retained more activity after lyophilization with or without the addition of cryopreservants, which did not produce a significant preservative effect.

Once greater retainment of PSI photoactivity than that with free PSI after lyophilization was confirmed, we next wanted to test the application of these microspheres in a biophotovoltaic device to assess their ability to perform in one of the imagined biotechnology applications of these particles. Biophotovoltaic devices were fabricated, and 500:1 BSA:PSI ratio microspheres were chelated overnight and then drop-coated on the semiconductor-coated electrode. A carbon-coated counter electrode and an aqueous synthetic cobalt-based electrolyte [42] were used to finish fabrication of the device.

**Fig. 6:**
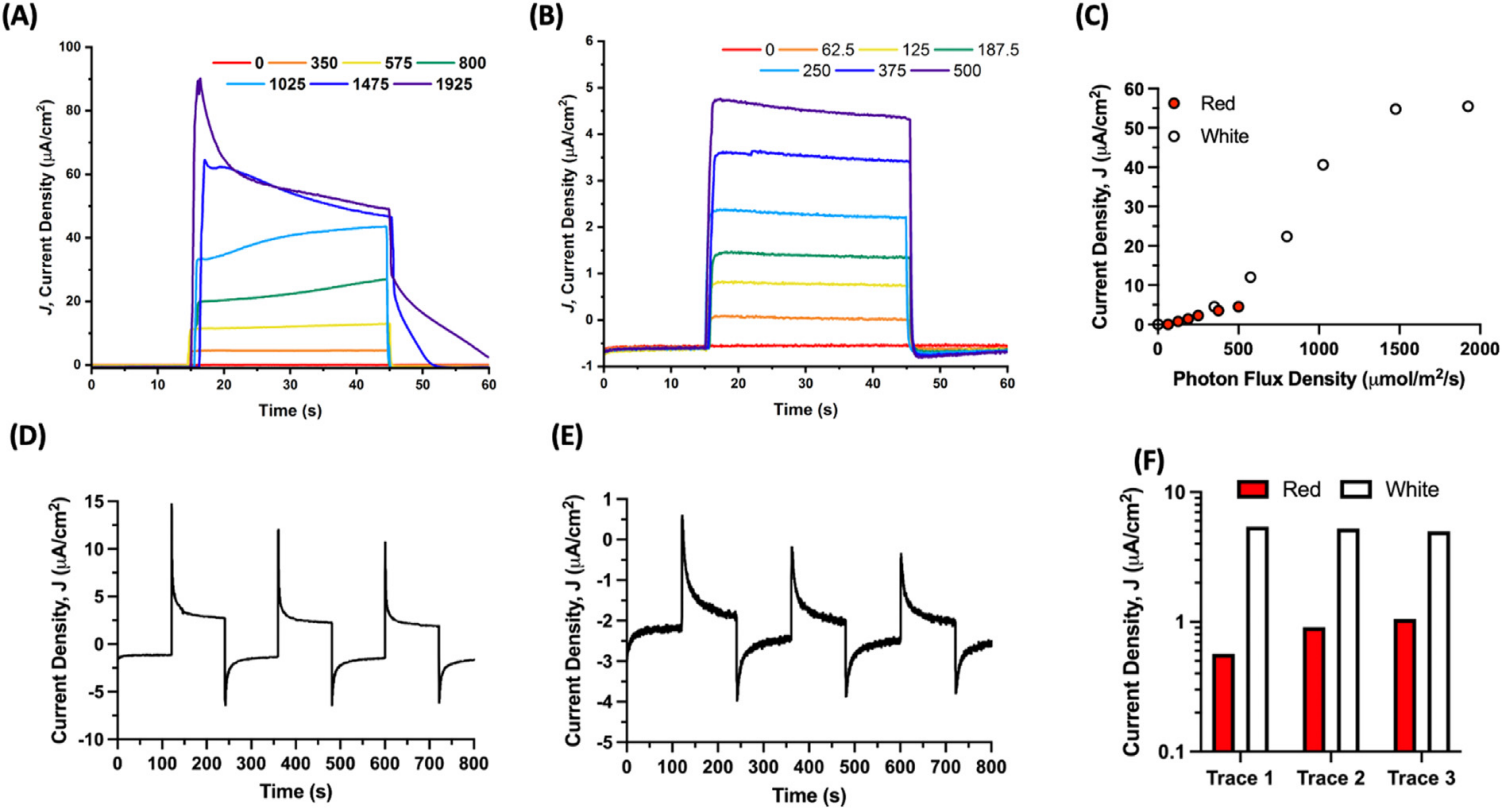
**PSI stabilized in microspheres is capable of generating photocurrent in a biophotovoltaic device.** PSI microspheres in a biophotovoltaic device are capable of undergoing charge separation and injecting electrons into a semiconducting TiO_2_ layer on an electrode. **A.** Photocurrent density dependence on light intensity of full-spectrum white light is reported. Light intensity is reported in photon flux density, PFD (μmol/m^2^/s). **B.** Photocurrent density dependence on light intensity for PSI-specific red actinic light is reported. Light intensity units are the same as panel A. **C.** Average photocurrent density was plotted against PFD to show the linearity of the response. **D, E.** Photocurrent density response is consistent over multiple light/dark cycles for both white (panel D) and PSI-specific red (panel E) light, respectively. The light intensity was 62.5 μmol photons/m^2^/s for the PSI-specific red actinic light and 350 μmol photons/m^2^/s for the broad-spectrum white light. **F.** The steady-state photocurrent density during light-on from panels D and E is quantified in panel F, with baseline current subtracted.

PSI microsphere–containing biophotovoltaic devices were capable of generating photocurrent under not only broad-spectrum white light but also red light specific for PSI photoexcitation, as seen in Fig. 6A and B. The PSI contained in microspheres was capable of constructive interaction via electron injection into the TiO_2_ semiconductor to allow for current to flow through the device. At 500 μmol photons/m^2^/s of PSI-specific red actinic light, the microsphere-containing photovoltaic device produced stable photocurrent densities of 5 μA/cm^2^. Under white light at nearly 2000 μmol photons/m^2^/s, photocurrent densities of ~60 μA/cm^2^ were able to be obtained, with an initial spike over 90 μA/cm^2^. Shown in Fig. 6C, this photocurrent produced was dependent on light intensity, with increasing photocurrent as the photon flux density increased. Under the broad-spectrum white light, there is a much steeper increase in photocurrent density generated at high light intensities before leveling off, likely due to the slight photoactivity of the TiO_2_ semiconductor itself.

The stability of PSI microspheres and their electronic output were then assessed over multiple light-dark cycles, shown in Fig. 6D and E. The steady-state photocurrent density output for each light/dark cycle was plotted with baseline dark current subtracted in Fig. 6F. Under white light, the steady-state photocurrent density produced was stable over multiple cycles and did not degrade. Interestingly, for the PSI-specific red light, the photocurrent density output increased between all traces, suggesting that PSI contained in the microspheres in the device was able to more efficiently fill the electron holes of the semiconductor, resulting in greater photocurrent density yields.

## Conclusion

Biotechnology is an emerging area of research, and a growing industry worldwide, defined as the use of living organisms, systems, and processes for the benefit of society, industry, and the environment [51]. The United States is the current world leader in the development of a biotechnology industry, with annual revenues from companies totaling over $300 billion USD in 2012. This is estimated to further grow over 10% each year, making it one of the fastest growing economic areas [52]. However, there are numerous challenges to continued growth and development of novel biotechnology applications, many of which are related to longevity and stability of the biomaterial of interest [53,54]. More generally, many biotechnologies rely on the use of proteins, which are susceptible to aggregation, degradation, and loss of activity along with issues of long-term storage exacerbating many of those issues. PSI specifically has been shown to have promise for the development of bioelectronic electrodes and biohybrid photovoltaic devices. There has been some work interested in the stabilization of PSI for biotechnology applications, including design of designer surfactant peptides and fully aqueous electrolyte systems, but the successful stabilization of proteins for long-term usage *in vitro* remains a burgeoning research area [42,53,54]. Herein, we describe applications of calcium carbonate microspheres, CCMs, as templates for the encapsulation of the protein-pigment complex PSI, forming aqueous, mesoporous non-aggregating proteinaceous microspheres. CCMs have been described previously as drug delivery vehicles in the pharmaceutical industry because of their biocompatible nature and ability to be facilely functionalized and modified [5]. We modified existing synthesis protocols for CCMs to accommodate the encapsulation of cyanobacterial PSI, a membrane bound protein-pigment complex while retaining photoactivity and improving its long-term storage capabilities.

We have developed a method of synthesizing and using CCMs to encapsulate PSI without loss of structural stability or protein activity, which can likely be modified as needed for other membrane proteins. This synthesis protocol yields highly uniform particles in both size distribution and protein distribution within the fabricated microspheres. The size of these microspheres was found to be dependent on both length of salt precipitation during initial formation and on the amount of inert filler protein added as well. These uniform particles are also non-aggregating and monodispersed, making them ideal candidates for use in flow cytometry or microfluidics applications. Chelation with EDTA after cross-linking of the protein of interest with filler BSA went essentially to completion, leaving behind proteinaceous mesoporous microspheres. After chelation to remove the CaCO_3_ template, we have shown that the final mesoporous microspheres are porous and their interiors are accessible to small molecules, as our PSI-containing particles are able to be exogenously reduced by treatment with DCPIP. This may prove beneficial for further biopharmaceutical development to help target activation of encapsulated proteins after delivery.

Although some fraction of total PSI photoactivity was lost during the encapsulation and fabrication process, the majority was retained. This further suggests the structural integrity of the membrane protein encapsulated was retained, as PSI utilizes a complex series of internal cofactors for electron transfer and must coordinate a large chlorophyll antenna that is highly specifically placed in the complex for optimal light-harvesting capabilities. The encapsulated PSI also proved to be more resistant to freeze-thaw stress and the lyophilization process, retaining a greater fraction of its photoactivity than free PSI. Most interestingly, the synthesized PSI microspheres maintained their photoactivity when integrated into a biophotovoltaic device. This produced photocurrent was dependent on the light intensity as expected, with increasing photocurrent density as light intensity increased. Photocurrent was produced even under PSI-specific red actinic light, suggesting that this photocurrent is being driven from the encapsulated reaction center. The biophotovoltaic devices were also stable over multiple traces, with no loss of photocurrent under broad-spectrum white light and a slight increase under the PSI-specific light. This may be due to PSI filling more of the electron holes of the semiconductor over time. This device design confirms the ability of PSI to simultaneously inject electrons into the TiO_2_ semiconductor and also accept electrons from the water soluble, Co^+2^ electron donor. These concurrent properties are most probably derived from the mesoporous nature of the microspheres, confirm the ability of PSI to interact very closely with the semiconducting material, and potentially demonstrate the ability for exciton transfer between adjacent, cross-linked PSI complexes. The role of BSA acts as spacer protein yet does not seem to block electron transfer at either surface or act as an insulator, potentially driving the photocurrent down or preventing forward electron injection from the PSI into the TiO_2_ semiconductor. Future work will try other spacer proteins besides BSA to further optimize the composition and porosity of the microspheres for future bioelectronics applications, and other avenues of interest include addition of various light-harvesting antennas to increase the wavelengths of light available for PSI photoexcitation.

Taken together, this work suggests that CCM encapsulation may be a viable strategy for improved activity, longevity, and storage of membrane proteins of interest for various biotechnology applications. This is, to our knowledge, not only the first report of a membrane protein being encapsulated utilizing CCMs but also the largest protein to be stabilized by this method. This is also, to our knowledge, the first report of microsphere-encapsulated proteins being incorporated into a biohybrid electronic device.

## Credit author statement

AHT and BDB conceptualized this research. AHT, LBT, SV, MC, and GL all aided in investigation and performing experiments. AHT, LBT and BDB were involved in writing and editing of the manuscript. AHT, LBT, MC and SV all helped in visualization of data. BDB provided supervision and funding acquisition to support this research.

## Declaration of competing interest

The authors declare that they have no known competing financial interests or personal relationships that could have appeared to influence the work reported in this paper.
